# A Dictionary of Practical Medicine, Comprising General Pathology, the Nature and Treatment of Disease, Morbid Structure, &c.

**Published:** 1845-03

**Authors:** 


***A Dictionary of Practical Medicine,*** comprising General Pathology, the Nature and Treatment of Disease, Morbid Structure, &c. By James Copeland, M. D., F. R. S. Edited, with additions, by Charles A. Lee, M. D. Part 4th. Harper & Brothers, New York.

This excellent dictionary furnishes, in a convenient form, and at a cheap rate, a valuable book of reference for the physican. While busily engaged in professional practice, he often feels the necessity of having at hand a compendium of modern medicine, to which he can refer for information and advice, and, indeed, very often for comfort; for nothing is more soothing to the vexed spirit of an anxious or baffled physician, than to be assured, by competent authority, that he is doing, or has done, all that can or could be done for the relief of his patient. In the hurry of practice, it is often impossible to search a volume for the information he wants, and, therefore, it is of priceless importance to have such a dictionary as Dr. Copeland's at hand. Every country practitioner, especially, should possess a copy of it.

He cannot always have access to so good a consulting physician as Dr. Copeland, and to one, of whom he can have so little jealousy. Dr. C. has embodied in this work the results of thirty years' practice, and the fruits gathered from most extensive reading.

The additions by the American editor, enhance very greatly its value. We strongly recommend the work.—*Balt. Ed.*

